# Supplementary material for: Room-temperature multistage metastability in a moiré superstructure
Source: Nat Commun. 2026 Jun 2;17:7123. doi: 10.1038/s41467-026-73482-6 (PMC13396654; doi:10.1038/s41467-026-73482-6)
Supplement: Supplementary file 1 — Supplementary Information [file 41467_2026_73482_MOESM1_ESM.pdf]

Supplementary Information for "Room-temperature multistage metastability in a moiré superstructure"

Baiqing Lv,[1,2,*] Yifan Su,[2,*] Alfred Zong,[2,3,4,*] Karna Morey,[2,3] Bryan T. Fichera,[2] Qiaomei Liu,[5] Dong Wu,[6] Yongchang Ma,[7] Dupeng Zhang,[1] Faran Zhou,[8] Makoto Hashimoto,[9] Dong-Hui Lu,[9] Donald A. Walko,[8] Haidan Wen,[8,10] Jiarui Li,[4,11] Suchismita Sarker,[12] Jacob P. C. Ruff,[12] Nanlin Wang,[1] and Nuh Gedik[2,†]

[1] *Tsung-Dao Lee Institute, School of Physics and Astronomy, Shanghai Jiao Tong University, Shanghai 200240, China.*

[2] *Massachusetts Institute of Technology, Department of Physics, Cambridge, Massachusetts 02139, USA.*

[3] *Department of Physics, Stanford University, Stanford, California 94305, USA.*

[4] *Department of Applied Physics, Stanford University, Stanford, California 94305, USA.*

[5] *International Center for Quantum Materials, School of Physics, Peking University, Beijing 100871, China.*

[6] *Beijing Academy of Quantum Information Sciences, Beijing 100913, China.*

[7] *School of Materials Science and Engineering, Tianjin University of Technology, Tianjin 300384, China.*

[8] *Advanced Photon Source, Argonne National Laboratory, Lemont, Illinois 60439, USA.*

[9] *Stanford Synchrotron Radiation Lightsource, SLAC National Accelerator Laboratory, Menlo Park, California 94025, USA.*

[10] *Materials Science Division, Argonne National Laboratory, Lemont, Illinois 60439, USA.*

[11] *Stanford Institute for Materials and Energy Sciences, SLAC National Accelerator Laboratory, Menlo Park, California 94025, USA.*

[12] *Cornell High Energy Synchrotron Source, Cornell University, Ithaca, New York 14853, USA.*

CONTENTS

I. Optical image of sample device 2

II. Complete circuit design 2

A. Pulsed voltage generator 3

B. Resistance measurements 4

C. Switching between two circuits 5

III. Temperature dependence of voltage-induced metastable states 6

IV. X-ray reciprocal space mapping images 7

V. Energy distribution curve analysis and reproducibility of ARPES data 9

VI. Estimation of Joule heating from excitation pulses 11

VII. Recovery timescale of the pulse-induced CDW states 12

Supplementary references 14

I. OPTICAL IMAGE OF SAMPLE DEVICE

Figure S1 demonstrates the geometry of a typical $EuTe_4$ device for in-situ measurements at synchrotron light sources for both XRD and ARPES. The black part in the center of the image is a cleaved crystal of $EuTe_4$. Electrical contacts are made with silver epoxy.

For XRD measurements, the $EuTe_4$ devices are fabricated on amorphous glass cover-slides so that the substrate only contributes a negligible diffuse background to the XRD measurements.

II. COMPLETE CIRCUIT DESIGN

In this section, we provide a more detailed description of the circuit design that enables the pulsed-voltage injection and in-situ transport measurements on $EuTe_4$ devices. The

* These authors contributed equally: B.Q.L., Y.S., and A.Z.

† Correspondence to: gedik@mit.edu

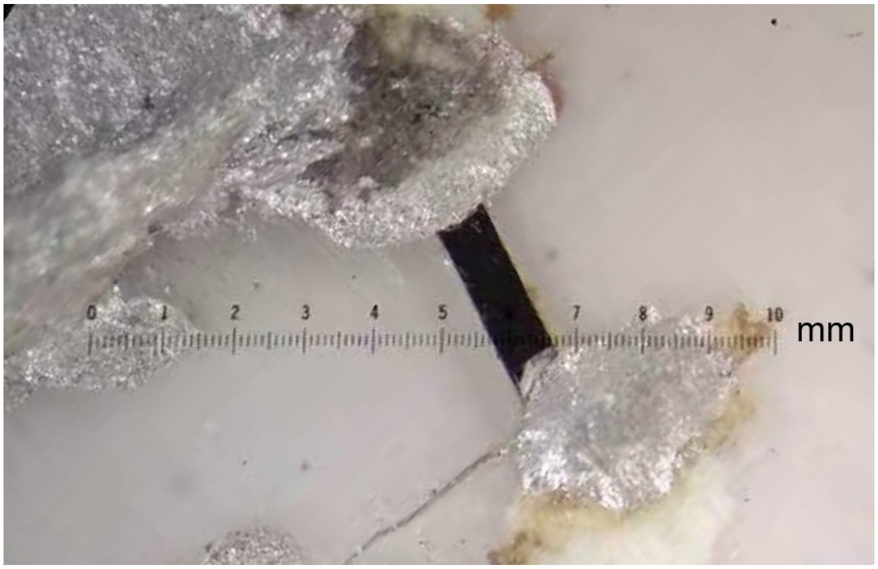

FIG. S1. **Optical image of sample device.** Optical micrograph of a typical $EuTe_4$ device for in-situ transport measurements at synchrotron light sources.

design is based on thesis work by K. Morey (Ref.[1]).

The circuit is designed to deliver a high voltage pulse (on the order of 10 to 15 V) to a small load between 50 and 150 Ω, meaning the circuit should be able to provide up to 300 mA of current to the sample. Additionally, the delivered pulse needed to be adjustable between 500 ns in duration and 100 ms with a rise time of no more than 100 ns. The system also needed to have electrical isolation between the resistance probe and the pulse generator so as to not introduce extra impedance from the pulse generator in the measurement of the sample resistance. Furthermore, due to the radiation safety requirements at high energy X-ray beamlines, all the components needed to be remotely controlled. To meet the requirements listed above, we developed a two-component system, with one component designed to deliver high voltage pulses to the sample, and another component designed to perform both short and long timescale based resistivity measurements. To keep the two components electrically isolated from each other, we used an electromechanical relay controlled by an Arduino.

A. Pulsed voltage generator

To provide a 10–15-volt pulse to a load with resistance between 50 and 150 Ω, we need to combine a traditional function generator, an operational amplifier, and current buffers.

We use a standard Agilent 33220A single-channel function generator to generate pulses of adjustable width between 500 ns and 100 ms at a repetition frequency of 0.3 Hz. The function generator ensures that a well-shaped pulse of a configurable height is sent once every three seconds into the rest of the system. However, this function generator and most others can only provide up to 10 V pulses and cannot provide more than 50 mA, meaning that voltage amplification and current boosting is necessary. To perform these two tasks, we use an operational amplifier in series with a current buffer, as show in Fig. S2

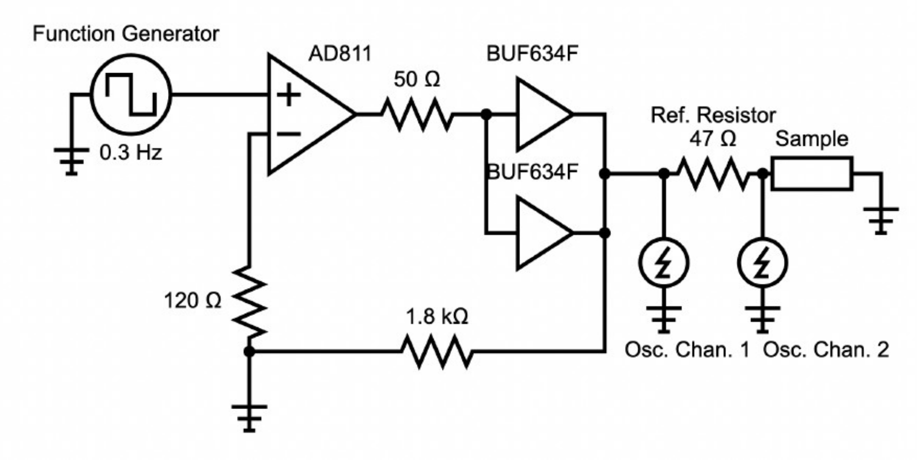

FIG. S2. **Circuit diagram of the pulse generator.** The circuit used for generating pulsed voltage and delivering it to $EuTe_4$ devices. BUF634F: current unity-gain buffer. AD811: Analog Devices operational amplifier

As traditional voltage feedback operational amplifiers cannot provide enough power to supply the amplified voltage to a small load of only 50 Ω, we thus use two BUF634F current unity-gain buffers, which have a high impedance input and provide an equivalent level of voltage but boosted current and power in the output terminal. The output of the current buffer is then connected to a 47 Ω reference resistor in series with the sample.

B. Resistance measurements

As also described in the main text, the circuit above in Fig. S2 is also capable of time-resolved resistance measurements during the pulsed voltage excitation via an oscilloscope. However, for a high-precision measurement of the sample resistance, seconds to hours after the application of the pulse, we need to use a Keithley ohmmeter, which has a much better

resolution compared to the oscilloscope.

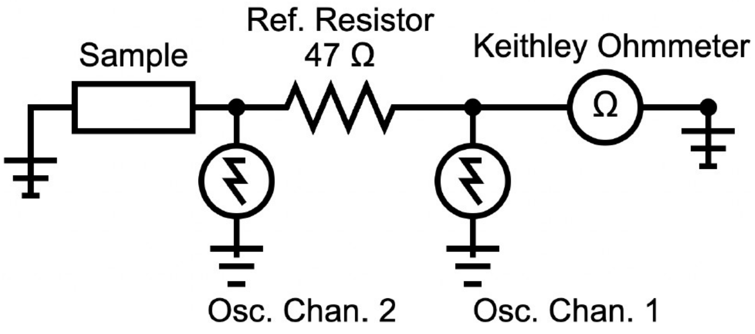

FIG. S3. **Circuit diagram of high-sensitivity resistance probe.** The circuit used for precisely measuring resistivity with a Keithley ohmmeter.

The circuit for high-precision resistivity measurement is shown in Fig. S3. The sample is connected in series with a reference resistor and the Keithley Ohmmeter. This circuit allows measurement of the resistance with high accuracy while isolating the sample from the voltage source between applied voltage pulses. We note that this design is also compatible with a cryogenic environment, as a minimum current is applied through the sample.

C. Switching between two circuits

The high-precision resistance probe circuit is fully isolated from the pulsed voltage circuit. To switch the sample between the two circuits remotely, depending on whether or not there is a pulse being applied, we use an electromechanical relay that acts as an electrically controlled switch[2]. Using an electromagnet, a sufficiently high voltage through the relay coil can magnetically alter the switch from the normally connected (NC) configuration to the normally open (NO) configuration, as shown in Fig. S4, while keeping two circuits completely isolated from each other.

The particular relay that we used was the Pasternack PE71S6392 radiofrequency electromechanical relay switch. A driver circuit is needed to drive the relay between the NC and NO configurations due to the high current needed to supply the 12 V relay operating voltage. Because the coil resistance of the relay is only around 22 ohms, the relay requires nearly 550 mA of current to turn on, which is much too high for standard microcontrollers

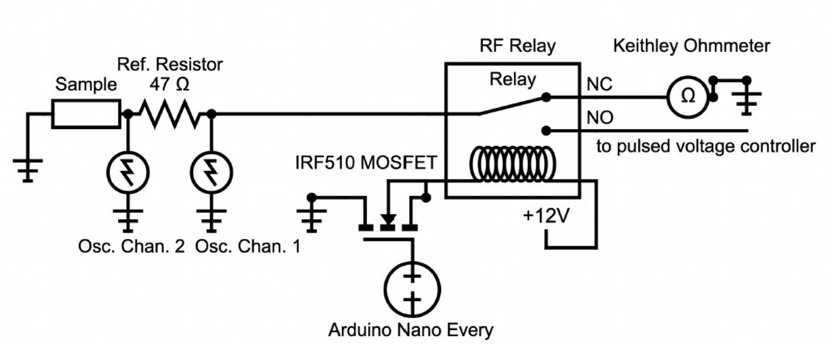

FIG. S4. **Design of switching mechanism.** A circuit diagram of the electromechanical relay and driver electronics used to electronically switch the relay between the high-precision resistnace probe circuit and pulsed voltage circuit. IRF510: N-channel metal–oxide–semiconductor field-effect transistor (MOSFET)

like the Arduino Nano Every. To accommodate the high current requirements, we use an IRF510 N-channel MOSFET transistor, which allows a very small gate voltage to control a much larger drain current, allowing a high amount of current to pass through the transistor drain, and, therefore, through the relay coils, as shown in Fig. S4.

Connecting the pulsed voltage circuit (Fig. S2) and the resistance probing circuit (Fig. S3) with the relay swiching circuit, the complete circuit diagram is shown in Fig. S5.

III. TEMPERATURE DEPENDENCE OF VOLTAGE-INDUCED METASTABLE STATES

The discussion in the main text focused on the measurements at 300 K in the heating branch of the thermal hysteresis loop. In this section, we discuss the temperature dependence of the voltage-induced metastable states.

The measurements at 400 K and 300 K in cooling branch are presented in Fig. S6. The results from 400 K is similar to those of 300 K in the heating branch presented in the main text. This is expected as the 400 K experiment is also taken after heating up the sample. However, in the cooling branch, we observed an increase in resistivity upon pulsed excitations. This suggests an enhancement in the CDW order parameter and thus the gap size. This is potentially an inverse process of what we discussed for the heating branch

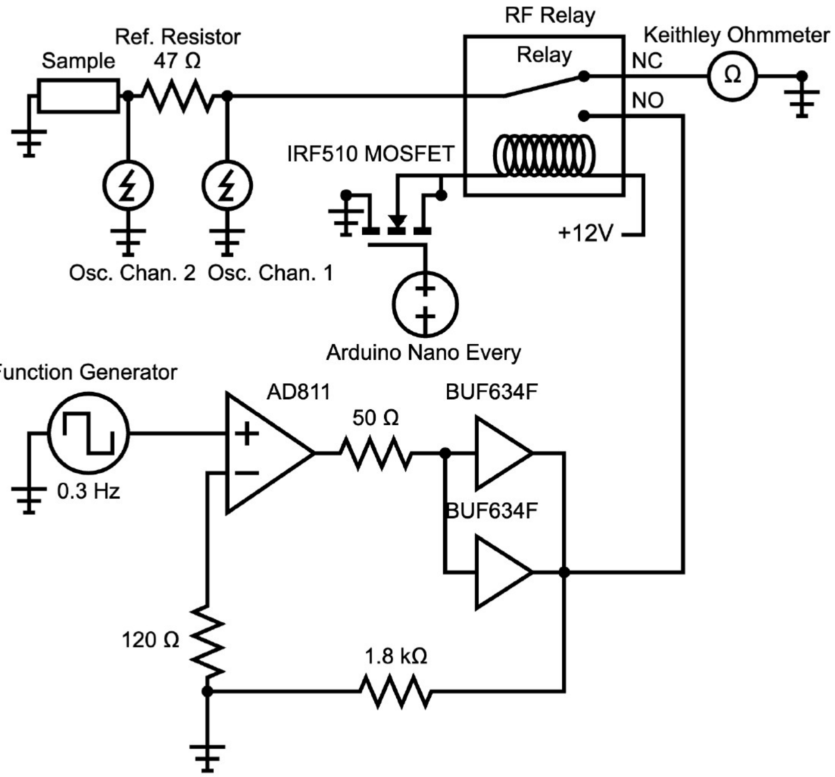

FIG. S5. **Full circuit diagram.** The complete system with pulsed voltage circuit and resistance probing circuit connected with relay switching circuit. BUF634F: current unity-gain buffer. AD811: Analog Devices operational amplifier. IRF510: N-channel metal–oxide–semiconductor field-effect transistor (MOSFET).

results. However, a detailed mechanism is subject to further investigation.

IV. X-RAY RECIPROCAL SPACE MAPPING IMAGES

In this section, we present the relevant cuts of XRD reciprocal space mapping data used for extracting information presented in Fig. 3 of the main text.

Figure S7 shows the (1 K L) cut of XRD reciprocal space mapping at 300 K in the heating branch measured at different timestamps in the pulsed voltage excitation process, as defined in Fig. 3. The XRD data are highly consistent with our previously reported results on $EuTe_4$ bulk crystals[3]. The relevant CDW satellite peaks are labeled in the first panel. The white arrows indicate monolayer CDW peak (q_1) and its second harmonic ($2q_1$) and the red arrow points to the bilayer CDW peak (q_2). As we prviously reported[3], other

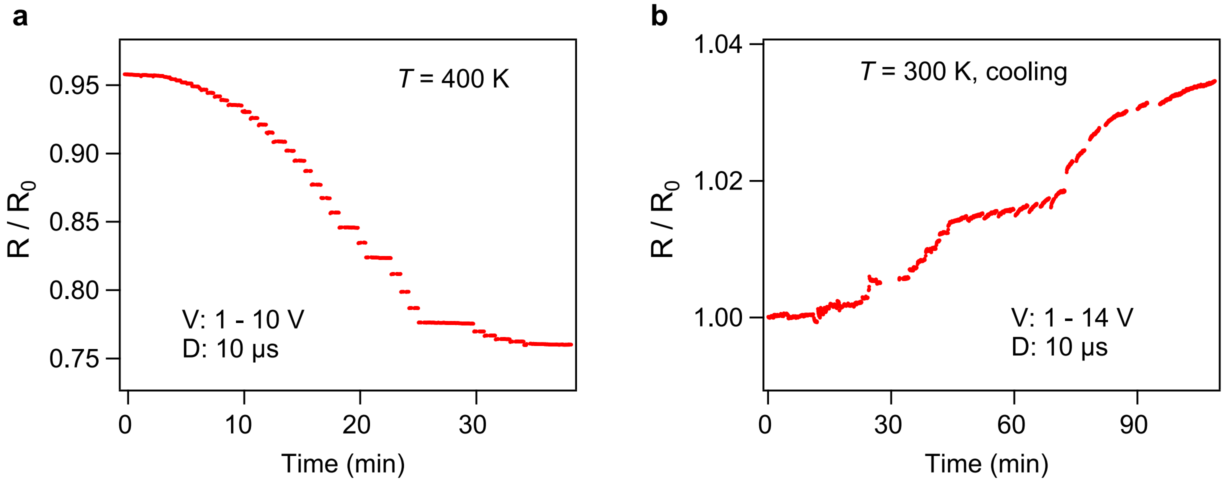

FIG. S6. **Resistivity upon pulsed excitation at different temperature points.** **a**, Normalized resistivity at 400 K, showing a similar pulse-induced decrease in resistivity similar to that observed at 300 K in the heating branch. **b**, Normalized resistivity at 300 K in the cooling branch, showing opposite behavior upon pulse excitation compared to the heating branch.

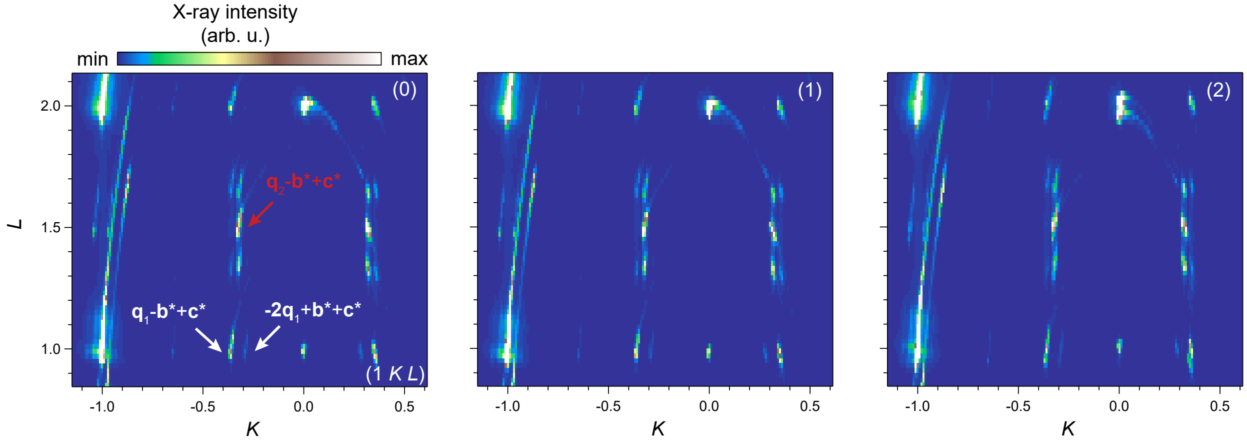

FIG. S7. **X-ray reciprocal space mapping images** (1 K L) cut of XRD reciprocal space mapping at 300 K in the heating branch. Labels (0)–(2) correspond to the timestamps before and after pulsed voltage excitation defined in Fig. 3a,b in the main text. Comparing the CDW satellite peaks in the images, a clear elongation along L direction together with a decrease in intensity is observed after pulsed voltage excitation.

non-integer peaks are present due to the stacking faults in bulk crystals and do not exist in a clean flake sample of $EuTe_4$ and thus can be safely neglected.

V. ENERGY DISTRIBUTION CURVE ANALYSIS AND REPRODUCIBILITY OF ARPES DATA

In Fig. 2 of the main text, we demonstrated the change of electronic structure in response to electrical pulse excitation via in-situ transport and ARPES measurements. In this section, we provide more detailed analysis and an additional dataset to prove the robustness of the ARPES results.

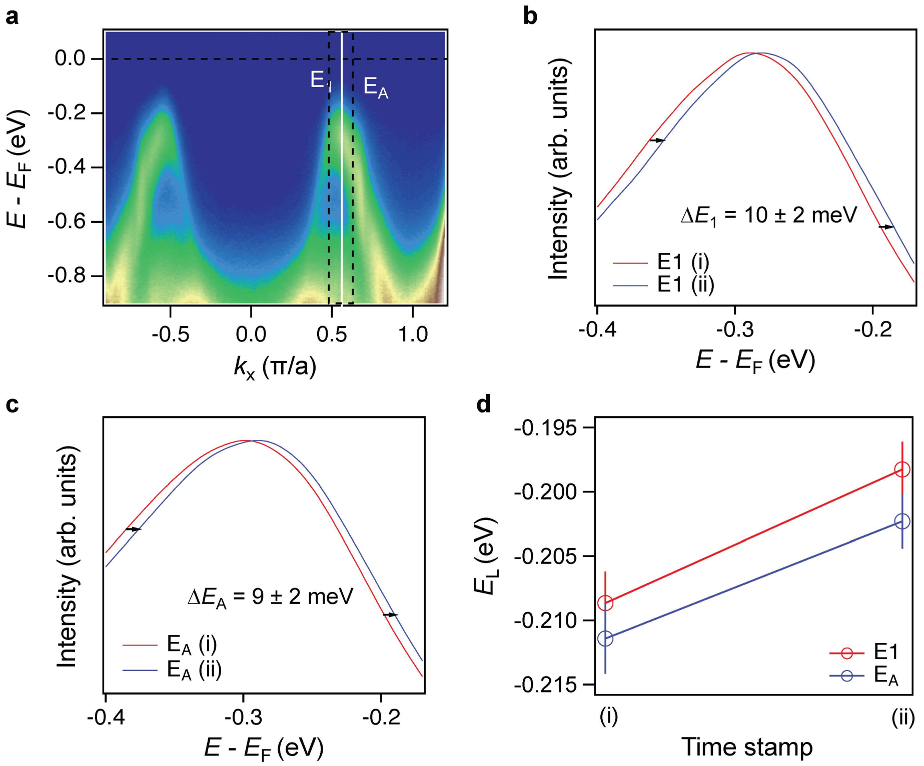

FIG. S8. **Robustness against the choice of momentum window for energy distribution curves study.** **a**, Energy-momentum dispersion cut of CDW-gapped band, same as Fig 2**b** in the main text. **b**, Energy distribution curves (EDCs) cut along the white solid line (E_1) in **a**, same as Fig. 2**e**. **c**, EDCs before and after pulse excitation acquired by integrating over the dashed momentum window in **a**. **d**, The leading edge positions before and after pulse excitation extracted from the EDCs with (E_A) and without (E_1) integrating over the momentum window. Showing consistent energy shifts, up to the systematic uncertainty of the measurement, in the band's leading edge.

In Fig. S8, we present additional EDC analysis on the data we demonstrated in Fig. 2 of

the main text. We show the robustness of our analysis by comparing the results in the main text (reproduced in Fig. S8**a-b**) to the EDCs integrated over a larger momentum window. In Fig. S8**c**, we show the EDCs integrated over the dashed window in Fig. S8**a**. Consistent with Fig. S8**b**, we observe the same energy shifts up to the systematic uncertainty of the measurement (2 meV), as indicated in the plot of band leading edges extracted from each of the EDC, shown in Fig. S8**e**.

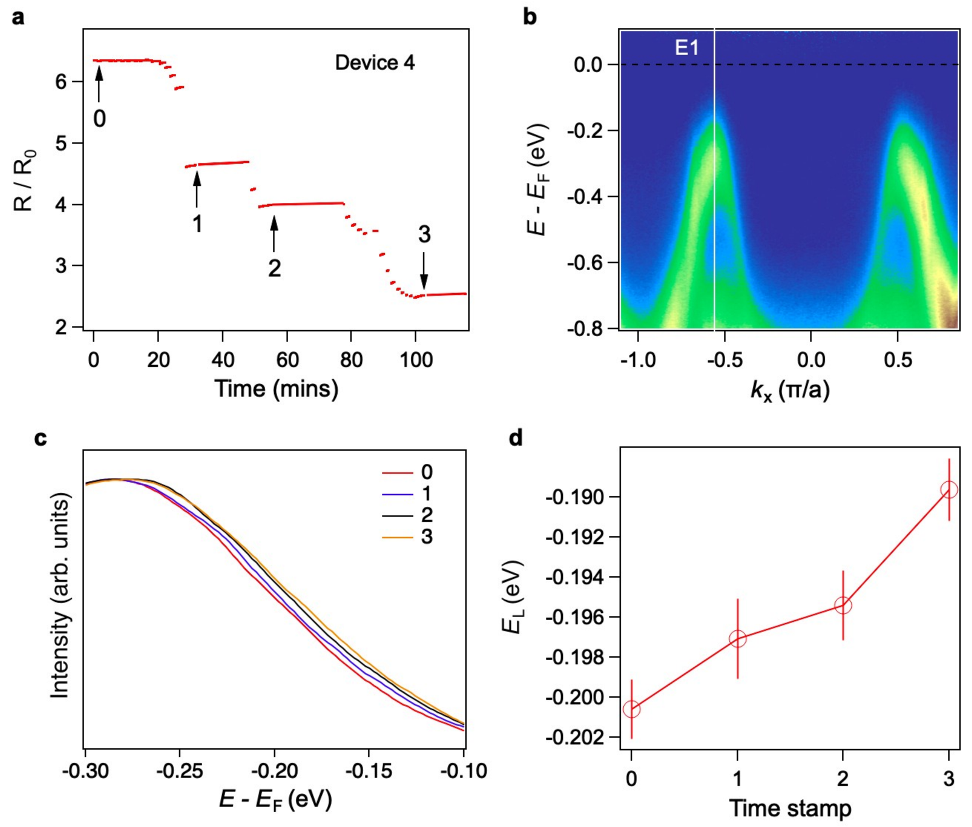

FIG. S9. **Reproduced CDW gap suppression upon current pulse excitation in ARPES measurement on another sample.** **a**, Concurrent resistance measurement upon pulse current excitation, showing decreasing resistance consistent to data presented in Fig. 2a. **b**, Energy-momentum cut of spectral function measured with ARPES on new device, consistent with the spectrum in Fig. 2c. **c-d**, Energy distribution curves (**c**) and extracted leading-edge position (**d**) corresponding to the time stamps 0, 1, 2, and 3 in **a**, indicating a gradual rightward shift of the band leading edge and thus a suppression of CDW amplitude

In order to check the robustness and reproducibility of our ARPES experiment results, we further reproduced the ARPES and in-situ transport measurement in another device.

As shown in Fig. S9, we repeated the same experiment shown in the main text with extra ARPES checkpoints monitoring the electronic structures of the intermediate metastable states. Upon each pulse excitation, the leading edge of the CDW-gapped band gradually shifts towards the Fermi level, indicating suppression of the CDW gap, consistent with the observation presented in the main text.

VI. ESTIMATION OF JOULE HEATING FROM EXCITATION PULSES

In order to examine the effect of heating in the metastable CDW phases driven by current pulses, in this section, we provide a quantitative estimate of the possible Joule-heating effects associated with voltage pulses of different durations.

For simplicity, assuming that all electrical energy delivered by the voltage pulse is converted into heat within the sample before dissipating to the environment (neglecting the enthalpy associated with phase transitions and any possible energy emission at early times after current injection) and the entire sample is heated uniformly, the resulting temperature increase ΔT of the sample can be estimated as

$$\Delta T = \frac{E \times M}{C_p \times \rho \times V} \tag{S1}$$

where E is the electrical energy of the voltage pulse, $M = 662.4$ g/mol is the molar mass, C_p=125 J/(mol·K) is the molar heat capacity, $\rho = 6.768$ g/cm^3 is the mass density, and V is the volume of the $EuTe_4$ bulk crystal between the electrodes (assuming the entire sample is homogeneously heat up).

Unlike the cases in previous studies[4,5], which focus on thin flakes, our measurements are performed on bulk single crystals. A typical sample volume is approximately $2 \times 0.5 \times 0.03$ mm^3, corresponding to $V \approx 3 \times 10^{-5}$ cm^3. Using these parameters, the effective heat capacity of the probed crystal volume is estimated to be on the order of 3.83×10^{-2} J/K.

As a representative example, we consider Device 2 in the heating branch at 300 K, with resistance $R \approx 500\ \Omega$, applied voltage $U = 12$ V, and pulse duration τ. The total electrical energy delivered by a pulse is estimated as

$$E = \frac{U^2 \tau}{R} \tag{S2}$$

For a pulse with a duration of 10 µs, this yields $E \approx 2.88 \times 10^{-6}$ J, corresponding to a temperature increase of $\Delta T \approx 0.075$ K. Even for a much longer pulse duration of 1 ms,

the estimated temperature increase is only approximately 7.5 K, which corresponds to a resistance change of approximately 8% (see Fig. 1**b**). This is below the observed drop of more than 50% (see Fig. 2**a**).

From the above analysis, we conclude that Joule heating is unlikely to be the dominant factor for short voltage pulses, particularly in the microsecond regime. However, it's also important to clarify that we cannot completely rule out thermal contributions, especially for longer pulses. Consistent with this consideration, the sensitivity of the metastable states to pulse duration may indicate that both electronic and thermal effects contribute to the observed metastability. Accordingly, we do not explicitly exclude thermal effects in the main text.

VII. RECOVERY TIMESCALE OF THE PULSE-INDUCED CDW STATES

In order to apply current-induced metastable states in $EuTe_4$ to electrical memory device, the metastable states need a long enough relaxation time so that the information storage is quasi-permanent. In this section, we examine the relaxation dynamics of resistance.

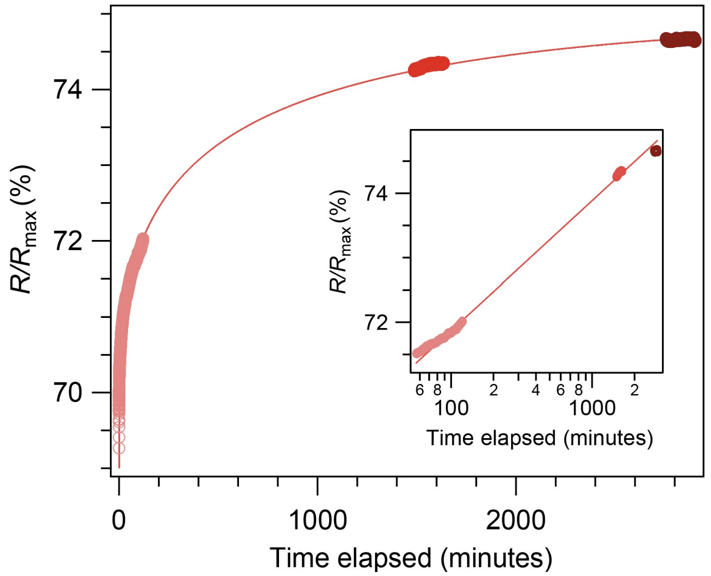

FIG. S10. **Recovery dynamics of resistance after pulsed current excitation**. Time evolution of resistance over the three days after applying current pulses. Inset: Same data plotted in logarithmic scale, featuring logarithmic recovery dynamics.

We performed resistance measurement over a timespan of over three days. As shown

in Fig. S10, after applying current pulses that reduce the resistance to around 70% of the original value, we can clearly observe an extremely slow logarithmic recovery. Extrapolating from the logarithmic fitting, the time for 60% resistance recovery (to 90% of the original value) is on the order of magnitude of 10^{11} hours (millions of years). We thus believe the switching can be considered as quasi-permanent.

SUPPLEMENTARY REFERENCES

[1] K. Morey, *Non-equilibrium dynamics in quantum materials*, Bachelor's thesis, Massachusetts Institute of Technology (2022).

[2] P. Horowitz and W. Hill, *The Art of Electronics*, 3rd ed. (Cambridge University Press, USA, 2015).

[3] B. Q. Lv, Y. Su, A. Zong, Q. Liu, D. Wu, N. F. Q. Yuan, Z. Nie, J. Li, S. Sarker, S. Meng, J. P. C. Ruff, N. L. Wang, and N. Gedik, Large moiré superstructure of stacked incommensurate charge density waves, Nature Materials **25**, 420 (2026).

[4] Q. Liu, D. Wu, T. Wu, S. Han, Y. Peng, Z. Yuan, Y. Cheng, B. Li, T. Hu, L. Yue, S. Xu, R. Ding, M. Lu, R. Li, S. Zhang, B. Lv, A. Zong, Y. Su, N. Gedik, Z. Yin, T. Dong, and N. Wang, Room-temperature non-volatile optical manipulation of polar order in a charge density wave, Nature Communications **15**, 8937 (2024).

[5] R. Venturini, M. Rupnik, J. Gašperlin, J. Lipič, P. Šutar, Y. Vaskivskyi, F. Ščepanović, D. Grabnar, D. Golež, and D. Mihailovic, Electrically driven non-volatile resistance switching between charge density wave states at room temperature (2024), arXiv:2412.13094.
