# Supplementary material for: Room-temperature multistage metastability in a moiré superstructure
Source: Nat Commun. 2026 Jun 2;17:7123. doi: 10.1038/s41467-026-73482-6 (PMC13396654; doi:10.1038/s41467-026-73482-6)
Supplement: Supplementary file 2 — Peer Review File [file 41467_2026_73482_MOESM2_ESM.pdf]

nature portfolio

Peer Review File

Room-temperature multistage metastability in a moiré superstructure

Corresponding Author: Professor Nuh Gedik

This file contains all reviewer reports in order by version, followed by all author rebuttals in order by version. Parts of this Peer Review File have been redacted as indicated to remove third-party material.

Version 0:

Reviewer comments:

Reviewer #1

(Remarks to the Author)
The authors report electrically induced metastable charge-density-wave (CDW) states in EuTe4 at and above room temperature. The manuscript presents an extensive set of measurements, including in-situ transport, ARPES, X-ray diffraction, and time-resolved resistance, which together form a high-quality and technically rigorous dataset. The ability to generate multi-level, nonvolatile metastable states over an exceptionally wide thermal hysteresis window is particularly impressive and represents a notable strength of the work. The experimental observations appear internally consistent and reveal a rich phenomenology that is scientifically noteworthy.

However, while the experimental results are compelling, the interpretation remains largely qualitative and would benefit from further clarification and quantitative support. The authors need to address the following issues:

1. The manuscript proposes that voltage pulses drive transitions among different out-of-plane CDW phase configurations. Yet no quantitative discussion is provided regarding how the applied electric field couples to these phase degrees of freedom, nor why such coupling would selectively modify the interlayer phases while the in-plane q-vectors, as shown by ARPES and XRD, remain unchanged. Without a physical mechanism or energetic argument describing this selective behavior, the present interpretation remains speculative.

2. The discrete resistance steps observed after repeated voltage pulses are an important aspect of the work, but it is not shown whether these steps correspond to specific CDW configurations or domain populations. The manuscript does not provide a quantitative relationship between resistance changes and the modifications observed in XRD peak intensity or broadening. As a result, the proposed association between the resistance levels and the possible phase-stacking configurations is not yet supported. In addition, the ARPES evidence for CDW gap suppression is based primarily on a small shift of a valence feature without direct information on the conduction-band minimum, making the inferred gap reduction uncertain. Clarification or additional supporting evidence would strengthen the claim.

3. The monolayer and bilayer CDWs respond differently to electrical pulsing, as reflected in the XRD measurements. The monolayer peaks are significantly more suppressed than the bilayer peaks, yet the manuscript does not provide a physical explanation for this asymmetry or indicate whether it is compatible with the proposed phase-stacking model. Discussion of whether differences in interlayer coupling, stiffness, or domain-wall energetics could account for this behavior would be valuable.

4. The time-resolved resistance data show a gradual decrease during each pulse, which the authors interpret as evidence for continuous field-driven evolution. However, resistance alone cannot exclude alternative possibilities such as partial CDW melting, or defect-assisted switching. A discussion of these limitations and of why structural dynamics are not essential for the proposed interpretation would improve the clarity of the argument.

5. The meaning of the schematic shown in Fig. 4(d) remains unclear. It is not evident whether the illustrated blocks represent real-space domain distributions, stacking sequences, or simply symbolic depictions of relative phase states. Because this figure plays an important conceptual role, its physical interpretation should be explicitly clarified.

In summary, the manuscript presents a rich and intriguing set of experimental findings, and the observation of robust

metastable states in EuTe4 is of genuine scientific interest. However, the microscopic interpretation requires further development, and establishing a clearer connection between the proposed model and the measured data would substantially strengthen the work. I would suggest major revision of the manuscript.

Reviewer #2

(Remarks to the Author)
The authors report in-situ transport, ARPES, and X-ray diffraction studies on bulk $EuTe_4$ crystals to investigate electrically induced metastable CDW states. Using in-situ voltage pulses, they observe multiple discrete resistance plateaus at room temperature, which they identify as nonvolatile metastable states within the material's large thermal hysteresis. ARPES measurements show a reduction of the CDW gap after pulsed excitation, while XRD shows decreased CDW peak intensities and shortened out-of-plane correlation lengths. Based on these observations, the authors propose that electrical pulses weaken the ground-state CDW and generate multiple types of CDW domains with modified phase configurations. They conclude that $EuTe_4$ hosts multistage, electrically tunable metastable CDW states suitable for high-temperature memory devices applications.

The observations are interesting and potentially relevant for memristor applications. However, the data that the authors presented can not fully support their claims. The similarities and differences between the current work and a highly relevant one, arXiv 2412.13094 (Ref. 30), are also not articulated well.

1) It is important to articulate the novelty of the current work in light of another study (Ref. 30), which also reported room-temperature, electrically switchable, metastable states in EuTe4. Moreover, this study also reported that the resistance of the metastable state is tunable by the applied voltage. However, Ref. 30 also mentioned that only a single pulse, with a duration down to tens of ns, is enough to saturate the resistance. This is inconsistent with the conclusions from the current study. The authors need to explain why their study is novel, and why their results can or cannot be reconciled with the previous study.

2) It is not experimentally supported that the resistance changes are due to only the reduction of CDW strength. In the manuscript, the author claimed that "electrical excitation induces disorder and potentially multiple domains, contributing to the observed sharp resistance drop." Yet in Fig.3c and d, from time stamp 0 to 1, the XRD measurements show essentially no reduction in either CDW intensity or out-of-plane correlation length, even though the resistance has already dropped significantly. The current manuscript does not provide an explanation for this discrepancy, which weakens the argument that CDW weakening directly drives the resistance change. Alternative mechanism or additional evidence should therefore be considered to substantiate this interpretation.

3) ARPES measurements at intermediate stages are needed. In Fig. 2, the authors present ARPES measurements only before and after the full sequence of electrical pulses. The rigid shift of the EDC before and after applying the electrical pulses was used as evidence for the suppression of the CDW gap in the metastable phase. However, ARPES measurement is missing at intermediate stages, unlike the XRD measurement in Fig. 3. If the resistance has already dropped significantly at a stage similar to time stamp 1, and if this drop indeed reflects partial suppression of the CDW order, one would expect a corresponding intermediate shift in the ARPES EDC. The absence of such measurements leaves a critical knowledge gap, and additional ARPES data would be necessary to clarify whether the electronic structure evolves stepwise in concert with the transport response.

In addition, the two CDW q-vectors should be labeled in the constant-energy contours in Fig. 2. It is not clear how to compare the ARPES results in the current study to a previous study by the same authors (Fig. 3 of Ref. 27).

4) The switching does seem to depend on the pulse duration, contrary to what the authors claimed. In Fig. 1c, the authors showed a voltage-dependent transport measurement, indicating a positive correlation between the applied voltage and the amount of resistance drop. However, in Fig. 4b, the magnitude of the resistance change also appears to grow systematically with increasing pulse duration, suggesting that both parameters, field strength and pulse duration, affect the transition. What the authors should do is two systematic studies: first, study the resistance after switching as a function of the applied voltage, fixing the pulse duration; second, study the resistance after switching as a function of the pulse duration, fixing the applied voltage. Without such systematic studies, no clear conclusion can be made.

5) In absence of the analysis separating the effects due to applied voltage and due to pulse duration, thermal contributions cannot be ruled out, especially given the well-documented large thermal hysteresis in $EuTe_4$. Both higher voltage amplitudes and longer pulses can transiently heat up the sample, which could independently lead to a decrease in resistance. One question is whether the authors tried a "heating" pulse. For example, one can apply such a heating pulse by applying a high repetition rate or cw laser. I note that an electrical pulse duration of micro-seconds is much beyond the typical time scale for lattice to be heated up.

6) The authors suggest that $EuTe_4$ could potentially be used as non-volatile memory devices. However, based on the proposed switching mechanism of gradual evolution of domain sizes, one would expect intrinsic stochasticity in the domain-evolution process. If structural domain growth governs the resistance changes, then after thermal erasure, repeated application of identical voltage pulses should not necessarily lead to identical resistance states. The authors should provide repeatability tests demonstrating that the resistance switching is reproducible over multiple cycles under identical electrical conditions. Without such evidence, the suitability of $EuTe_4$ as a reliable memory medium remains unproven.

7) Fig. 4d lacks appropriate labeling and explanation. What parameter space are these cubes plotted in? xyz space? kx-ky-kz space? It is not clear at all.

8) The paper only shows data in the heating cycle. What happens to the resistance switching in the cooling branch of the hysteresis loop (see Ref. 30)?

9) Resistances in the metastable states are still changing over time, even though it is slow. Note that if this is for quasi-permanent information storage, this would still be a big problem. The authors showed that the resistance changes by 7% in a few hours. What happens if we let the materials idle for a few days or a few weeks? Will the resistance completely recover to the value before switching?

Reviewer #3

(Remarks to the Author)
The manuscript by Lv et al. reports the observation of electrically driven multistage metastable states in $EuTe_4$ at room temperature, characterized by discrete resistivity plateaus and high sensitivity to the applied electric field. By integrating photoemission spectroscopy, X-ray diffraction, and transport measurements, the authors attribute these states to electric-field-induced switching between out-of-plane charge-density-wave (CDW) phases within the moiré superstructure.
The reported effect is interesting and may hold potential for memory device applications. The data analysis is technically sound in general. However, I have several major concerns regarding the novelty of the findings relative to very recent literature, as well as the interpretation of key experimental results, which must be addressed before the manuscript can be considered for publication.
1. Two preprints/papers (Nature Communications 15, 8937 (2024) and arXiv:2412.13094) also reported nonvolatile metastable states in $EuTe_4$, induced by light and electric field, respectively. The authors must explicitly clarify the principal advance of the present manuscript compared to these works. A more detailed and focused discussion highlighting the distinct mechanism (e.g., the specific role of out-of-plane CDW switching in a multi-stage manner), or new insights offered here, should be added to the introduction and/or discussion.
2. Is the electric-field-induced resistance switching observable in the normal phase of $EuTe_4$ (i.e., above the CDW transition temperature), or is it exclusively enabled by the presence of the CDW order? A control experiment above the transition temperature would be crucial to establish the essential role of the CDW state and strengthen the proposed mechanism.
3. The in-situ ARPES data in Fig. 2d, intended to demonstrate CDW melting via a band shift after voltage pulses, does not show a visually clear energy shift. Furthermore, only one energy distribution curve (EDC) is presented in Fig. 2e. Could the authors provide a more quantitative analysis (e.g., momentum-integrated EDCs) to unequivocally demonstrate a rigid shift or CDW gap melting?
4. Please state the energy resolution and measurement temperature of the ARPES experiment, as these parameters are critical for interpreting subtle spectral changes.
5. The X-ray diffraction data in Fig. 3 shows a significant change in resistivity after the first voltage pulse, yet the corresponding diffraction peak intensity and full width at half maximum (FWHM) appear nearly unchanged. Are there additional quantitative data (e.g., integrated intensity and FWHM fitting results) to substantiate the claimed evolution?
6. The authors conclude in page 8 that "the application of electric fields predominantly modifies the out-of-plane periodicity... while the in-plane periodicity remains preserved." What would be the effect if the electric field were applied along the out-of-plane direction (c-axis)? A discussion on the expected anisotropy based on the proposed mechanism is necessary to complete the physical picture.

Reviewer #4

(Remarks to the Author)
I co-reviewed this manuscript with one of the reviewers who provided the listed reports. This is part of the Nature Communications initiative to facilitate training in peer review and to provide appropriate recognition for Early Career Researchers who co-review manuscripts.

Version 1:

Reviewer comments:

Reviewer #1

(Remarks to the Author)
The authors have addressed all my concerns. I recommend the manuscript for publication.

Reviewer #2

(Remarks to the Author)
The authors have done a commendable job in responding to questions from me and other reviewers. The insight about how the switching dynamics is different for bulk samples and for thin flakes with 10s of nm thicknesses is very important. I recommend publication of this work.

Reviewer #3

(Remarks to the Author)
The authors have performed additional data analysis, and tried as much as possible to address the question regarding the nolvety of their work compared to two recent works. Although the novelty is not very obvious, this work has included more detailed measurements, providing useful information for understanding the driven metastable phase in EuTe4.

Reviewer #4

(Remarks to the Author)
I co-reviewed this manuscript with one of the reviewers who provided the listed reports. This is part of the Nature Communications initiative to facilitate training in peer review and to provide appropriate recognition for Early Career Researchers who co-review manuscripts.

Open Access This Peer Review File is licensed under a Creative Commons Attribution 4.0 International License, which permits use, sharing, adaptation, distribution and reproduction in any medium or format, as long as you give appropriate credit to the original author(s) and the source, provide a link to the Creative Commons license, and indicate if changes were made.
In cases where reviewers are anonymous, credit should be given to 'Anonymous Referee' and the source.
The images or other third party material in this Peer Review File are included in the article’s Creative Commons license, unless indicated otherwise in a credit line to the material. If material is not included in the article’s Creative Commons license and your intended use is not permitted by statutory regulation or exceeds the permitted use, you will need to obtain permission directly from the copyright holder.
To view a copy of this license, visit https://creativecommons.org/licenses/by/4.0/

Reviewer #1 (Remarks to the Author):

The authors report electrically induced metastable charge-density-wave (CDW) states in EuTe4 at and above room temperature. The manuscript presents an extensive set of measurements, including in-situ transport, ARPES, X-ray diffraction, and time-resolved resistance, which together form a high-quality and technically rigorous dataset. The ability to generate multi-level, nonvolatile metastable states over an exceptionally wide thermal hysteresis window is particularly impressive and represents a notable strength of the work. The experimental observations appear internally consistent and reveal a rich phenomenology that is scientifically noteworthy.

We thank the reviewer for acknowledging the quality of the study and the value of the discovery.

However, while the experimental results are compelling, the interpretation remains largely qualitative and would benefit from further clarification and quantitative support. The authors need to address the following issues:

1. The manuscript proposes that voltage pulses drive transitions among different out-of-plane CDW phase configurations. Yet no quantitative discussion is provided regarding how the applied electric field couples to these phase degrees of freedom, nor why such coupling would selectively modify the interlayer phases while the in-plane q-vectors, as shown by ARPES and XRD, remain unchanged. Without a physical mechanism or energetic argument describing this selective behavior, the present interpretation remains speculative.

We thank the reviewer for the question. We would like to first reiterate the unique joint lock-in phenomenon in the CDWs in $EuTe_4$. As we discovered in our previous study [1], the two CDWs in monolayer and bilayer Te sheets forms a pair of jointly commensurate CDW (Fig. R1). This joint lock-in makes the *q*-vector extremely robust, not only against temperature, but also against optical excitation, as we revealed in a more recent ultrafast pump-probe study [2], as well as electrical excitation as we discuss in the present work. As a result of the joint lock-in, the energy barrier of changing *q* vectors is much higher than shifting the relative interlayer phases.

[Figure Redacted]

Figure R1. **Jointly commensurate CDWs in $EuTe_4$**. The q-vectors of monolayer and bilayer CDWs in EuTe4 satisfies the joint commensuration condition $q_1+2q_2 = 2b^*$ (lower panel). This lock-in phenomenon makes the q-vector extremely robust across a large temperature range (upper panel). Reproduced from Ref. [1].

According to a recent theoretical work [3], the ground state configuration has the lowest energy. The difference between the ground state and metastable states is on the order of tens of milli-electronvolts, with the highest energy state being $\theta_1 = 0$, $\theta_2 = \pi$,$\theta_3 = 0$, where $\theta_{1,2,3}$ are defined in Fig. 4c, with an energy difference of 12.6 meV (Fig. R2). While pump-probe suggests that the energy barrier for shifting q vector is way beyond 1 eV [2]. We can thus conclude that interlayer phase shift is an energetically favorable scenario.

[Figure Redacted]

Figure R2. **DFT calculation results for the energies of distinct CDW configurations**. Each state (*S*) is l abelled by three parameters, each corresponding to the CDW distortion in one of three Te square net sheet s within one unit cell. Each configuration is composed of two states in adjacent unit cells. The difference between the highest-energy metastable state and the ground state is 12.6 meV. Reproduced from Ref. [3].

Changes made:

We supplemented the discussion about the energy difference between the ground state and metastable states with approximate calculation results. We also highlighted the robustness of in-plane *q*-vectors against thermal and optical excitations due to joint lock-in in the introduction paragraphs.

2. The discrete resistance steps observed after repeated voltage pulses are an important aspect of the work, but it is not shown whether these steps correspond to specific CDW configurations or domain populations. The manuscript does not provide a quantitative relationship between resistance changes and the modifications observed in XRD peak intensity or broadening. As a result, the proposed association between the resistance levels and the possible phase-stacking configurations is not yet supported. In addition, the ARPES evidence for CDW gap suppression is based primarily on a small shift of a valence feature without direct information on the conduction-

band minimum, making the inferred gap reduction uncertain. Clarification or additional supporting evidence would strengthen the claim.

We agree with the reviewer that the discrete resistance steps are an important observation as it indicates a finite number of CDW layers or domains that has been switched. We would like to first point out that in Fig. 3d, we demonstrated the FWHM of diffraction peaks along *c* direction. This is a direct evidence of decrease in correlation length, and thus increase in domain population. Combining with the simultaneous resistance measurement presented in Fig. 3a, we believe this provides a strong evidence that supports the pulse-induced decrease in resistivity and increase in domain population.

In terms of specific CDW configurations, we would like to point out that probing the stacking configuration in the 3D domain structure in real space is beyond the capability of simple X-ray scattering. Imaging the cross-sectional CDW configuration in *b*-*c* crystalline plane would need much more complicated experimental techniques such as 4D-STEM ptychography or coherent X-ray diffraction imaging [4,5]. Even with such a technique, measure the complete configuration space requires a large number of samples and involved algorithms. We thus believe this task is beyond the scope of the present work.

We agree that direct measurement of the full CDW gap would be ideal. However, the conduction band minimum lies ~0.2 eV above the Fermi level, making it inaccessible to equilibrium ARPES and the only method to access the full CDW gap is time-resolved ARPES (trARPES) with ultrafast pump-probe capability, which we performed previously [6]. However, such instruments are so far not compatible with in-situ transport measurements and thus are beyond the scope of this work. Instead, our key evidence for CDW weakening comes from the upshift of the valence band top and the concurrent decrease in superlattice peak intensity (Fig. 3c).

It is also worth mentioning that closely following Reviewer #2's suggestions, we also performed additional *in situ* ARPES and transport measurements at intermediate stages of the voltage-pulse sequence. The main results are summarized in Fig. R3. As correctly noted by the reviewer, a gradual upward shift of the EDC leading edge is observed, which correlates well with the concurrent change in resistance. This consistency provides further evidence for a progressive suppression of the CDW gap in the metastable phase.

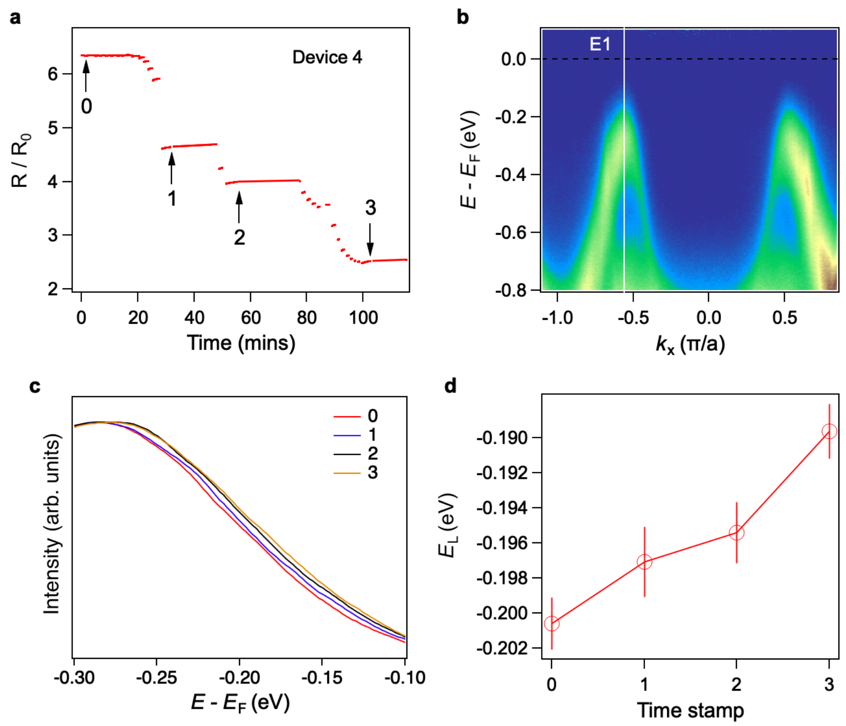

Figure R3. **Reproduced CDW gap suppression upon current pulse excitation in ARPES measurement on Device 4. a**. Concurrent resistance measurement upon pulse current excitation, showing decreasing resistance consistent with data presented in Fig. 2a. **b**. Energy-momentum cut of spectral function measured with ARPES on device 4, consistent with the spectrum in Fig. 2c. **c-d**. Energy distribution curves (**c**) and extracted leading-edge position (**c**) corresponding to the time stamps 0,1,2, and 3 in **a**, indicating a gradual upward shift of the band leading edge and thus a suppression of CDW amplitude.

Changes made:

We explained the relationship between the discrete resistivity plateau and the CDW domain switching in the discussion of metastable state formation. We also added a new section in the supplementary information for additional transport-ARPES measurement results.

3. The monolayer and bilayer CDWs respond differently to electrical pulsing, as reflected in the XRD measurements. The monolayer peaks are significantly more suppressed than the bilayer peaks, yet the manuscript does not provide a physical explanation for this asymmetry or indicate whether it is compatible with the proposed phase-stacking model. Discussion of whether differences in interlayer coupling, stiffness, or domain-wall energetics could account for this behavior would be valuable.

We appreciate this close inspection. Indeed, compared to bilayer CDW, monolayer CDW has a smaller gap, thus smaller order parameter amplitude. and is thus more susceptible to external perturbation. This was shown in the previous trARPES study [6]. Upon photoexcitation, the

monolayer CDW gap is suppressed more and can eventually be fully closed at an elevated excitation fluence while the bilayer CDW gap remains open. We believe this hierarchy also applies to the case of electrical excitation. The different CDW amplitudes of monolayer and bilayer CDWs are not directly tied to the phase dynamics that governs the stacking of CDWs layers and predominantly concerns the out-of-plane energy landscape. Thus, there is no issue of compatibility between the distinct amplitude suppression and stacking phase shifts.

Changes made:

We have noted the reason for the difference in the suppression of monolayer and bilayer CDWs in the discussion of XRD data.

4. The time-resolved resistance data show a gradual decrease during each pulse, which the authors interpret as evidence for continuous field-driven evolution. However, resistance alone cannot exclude alternative possibilities such as partial CDW melting, or defect-assisted switching. A discussion of these limitations and of why structural dynamics are not essential for the proposed interpretation would improve the clarity of the argument.

We appreciate the plausible scenario proposed by the reviewer. Our original intention was to propose a simplest explanation, following the "Occam's razor principle," without involving complicated and speculative scenarios. However, we agree with the reviewer that defect-assisted switching is possible as well. The practical CDW melting can also be understood as the result of topological defect formation, similar to previous observations in time-resolved diffraction measurements. The topological defects would form a metallic network in the system [7]. This provides a plausible scenario for explaining the metastable low-resistance state.

Changes made:

We have reformulated the discussion on the driving mechanisms of resistance evolution. We have included other possibilities for completeness.

5. The meaning of the schematic shown in Fig. 4(d) remains unclear. It is not evident whether the illustrated blocks represent real-space domain distributions, stacking sequences, or simply symbolic depictions of relative phase states. Because this figure plays an important conceptual role, its physical interpretation should be explicitly clarified.

We thank the reviewer for pointing out the ambiguity of the schematics. The picture is meant to present the domain dynamics in real space. As shown in Fig. R4, the blue color represents the

domains with ground state CDWs, while other colors represent metastable domains, with each distinct color representing a distinct CDW stacking configuration.

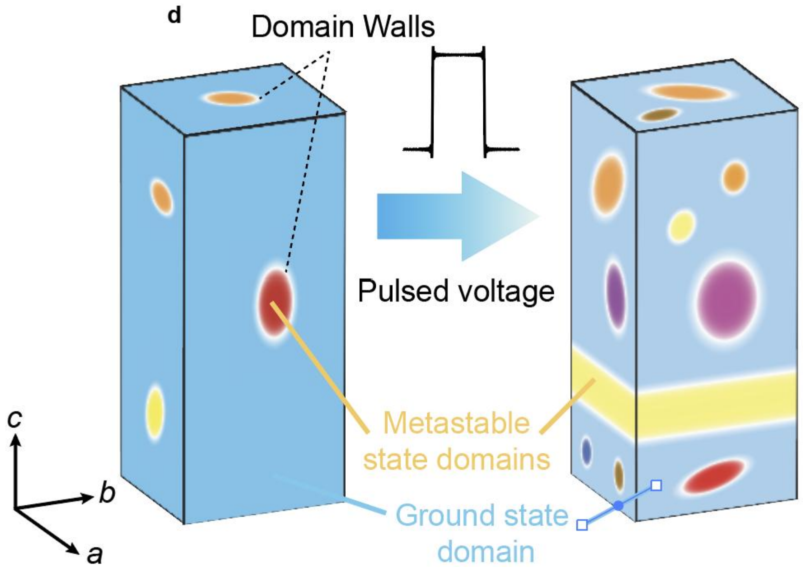

Figure R4. ***Revised Fig. 4d in the main text:*** **Schematics of voltage-induced metastable CDW states.** The cuboids indicate the domain dynamics in real space, labeled with crystalline axes. The area with cyan color represents the ground state, and areas with other colors represent metastable CDW domains, where different colors indicate different metastable phases with distinct relative phases between the Te layers, as color-coded in the table in **c**. The thin white boundaries indicate domain walls between distinct CDW domains. Upon pulsed electrical excitation, the ground state CDW amplitude is suppressed, and metastable CDW.

Changes made:

We have labelled spatial coordinates with crystalline axes. We have also added more detailed explanation in the caption as shown above in Fig. R4.

In summary, the manuscript presents a rich and intriguing set of experimental findings, and the observation of robust metastable states in EuTe4 is of genuine scientific interest. However, the microscopic interpretation requires further development, and establishing a clearer connection between the proposed model and the measured data would substantially strengthen the work. I would suggest major revision of the manuscript.

We thank the reviewer again for providing pertinent feedbacks on our manuscript. In the revised manuscript, we believe we addressed all questions from the reviewer by providing extra experimental data, more in-depth analysis, and more complete reasoning of physical mechanisms behind the pulse-induced metastable CDW states.

Reviewer #2 (Remarks to the Author):

The authors report in-situ transport, ARPES, and X-ray diffraction studies on bulk $EuTe_4$ crystals to investigate electrically induced metastable CDW states. Using in-situ voltage pulses, they observe multiple discrete resistance plateaus at room temperature, which they identify as nonvolatile metastable states within the material's large thermal hysteresis. ARPES measurements show a reduction of the CDW gap after pulsed excitation, while XRD shows decreased CDW peak intensities and shortened out-of-plane correlation lengths. Based on these observations, the authors propose that electrical pulses weaken the ground-state CDW and generate multiple types of CDW domains with modified phase configurations. They conclude that $EuTe_4$ hosts multistage, electrically tunable metastable CDW states suitable for high-temperature memory devices applications.

We sincerely thank the reviewer for their careful reading of our manuscript and for acknowledging the novelty and technical advances of our work.

The observations are interesting and potentially relevant for memristor applications. However, the data that the authors presented can not fully support their claims. The similarities and differences between the current work and a highly relevant one, arXiv 2412.13094 (Ref. 30), are also not articulated well.

We thank the reviewer for acknowledging the interest and relevance of our work for *Nature Communications*. Below we provide point-to-point answers to address specific concerns from the reviewer, especially the relationship between the present work and Ref. 30 (arXiv:2412.13094).

1) It is important to articulate the novelty of the current work in light of another study (Ref. 30), which also reported room-temperature, electrically switchable, metastable states in EuTe4. Moreover, this study also reported that the resistance of the metastable state is tunable by the applied voltage. However, Ref. 30 also mentioned that only a single pulse, with a duration down to tens of ns, is enough to saturate the resistance. This is inconsistent with the conclusions from the current study. The authors need to explain why their study is novel, and why their results can or cannot be reconciled with the previous study.

We appreciate this important point raise by Reviewer #2. As noted in our manuscript, recent transport and optical studies have demonstrated optical and electrical switching of hidden, nonvolatile CDW states in $EuTe_4$ flakes**,** opening the gateway towards understanding metastable behaviors in this room-temperature moiré superstructure (Refs. 29, 30).

The present work, however, is fundamentally distinct from these studies in platform, methodology, focus, and observed phenomena.

1. Material Platform. Refs. 29 and 30 focus on exfoliated $EuTe_4$ flakes with lateral dimensions of tens of micrometers and thicknesses of several tens of nanometers. In contrast, our study investigates bulk single crystals with typical dimensions of ~2×0.5×0.05 mm^3. This difference in dimensionality is crucial and leads to qualitatively different metastable behaviors.

2. Methodology and Focus. Ref. 29 focuses on light-induced metastable states, while Ref. 30 relies exclusively on transport measurements to reveal electrically switched states. In contrast, as illustrated in Fig. 2, our work uniquely combines *in situ* transport with ARPES and XRD to investigate current-induced metastable states. This integrated approach enables direct probing of CDW order parameters under voltage perturbation, and naturally shifts the focus of the present work towards revealing the nature and microscopic origin of the metastable states. We emphasize that performing *in situ* transport together with ARPES and XRD is technically nontrivial, requiring careful control of temperature, vacuum conditions, grounding, and data acquisition. To the best of our knowledge, this is the first work to integrate *in situ* transport, ARPES, and XRD within a single experimental framework.

3. Qualitatively Different Phenomena. As correctly noted by the reviewer, metastable behaviors in bulk crystals and flakes differ markedly (Fig.R5):

1. **Cooling branch:** For bulk crystals, the resistivity shows little change under voltage driving. In contrast, for flakes, a single voltage pulse of 2 V or an ultrafast optical pulse with fluence exceeding $F \sim 6.8$ mJ/cm^2 can drive the system into hidden high-resistance metastable states.
2. **Heating branch:** As correctly noted, metastable states in both bulk crystals and exfoliated flakes can be electrically tuned, but their responses to voltage pulses are distinct. In bulk crystals, a sequence of voltage pulses with increasing amplitude (up to 12 V) drives the system into multiple metastable states characterized by substantially reduced resistivity (exceeding a 50% drop). In contrast, for flakes, voltage pulses below 2 V induce metastable states with lower resistivity, whereas for voltages exceeding 2 V the system transitions into hidden high-resistance states.

These striking differences directly reflect the novelty and significance of the present work. As discussed in the main text, due to the quasi-two-dimensional nature of the Te layers, the in-plane couplings are much stronger than the out-of-plane couplings. Consequently, the application of an electric field predominantly modifies the out-of-plane periodicity of the monolayer and bilayer CDWs.

For simplicity, as illustrated in Fig. 4c, we assume that the intra-bilayer phases are locked ($\theta_3 = \pi$) and that the in-plane coherence length is effectively infinite. Under these assumptions, considering a $2c$ (~3.1 nm) unit yields four classes of out-of-plane CDW configurations: (0,0), (0,π), (π,0), and (π,π).

For thin flakes with typical thicknesses of several tens of nanometers, there are on the order of ten such units along the c-axis. A voltage pulse can therefore drive the entire stack into one of the metastable configurations, naturally explaining the emergence of hidden high-resistance states. In contrast, for bulk crystals with thicknesses of tens of micrometers, there are on the order

of 10^4 units along the *c* direction. It is therefore far more difficult to coherently align all units into a single metastable configuration. This naturally explains the observed distinct metastable behaviors of bulk crystals, especially the emergence of multiple metastable states in the heating branch and the absence of hidden high-resistance states in bulk crystals.

To summarize, our work is neither in contradiction with nor a reproduction of the findings in Refs. 29 and 30. Rather, it provides a complementary perspective. Specifically, the current work represents the first systematic study of current-induced metastable CDW phenomena in bulk $EuTe_4$ and the first to directly track voltage-driven evolution of CDW order parameters using a combined transport–ARPES–XRD approach. We therefore strongly believe that this study meets the scope and significance criteria of *Nature Communications*.

[Figure Redacted]

Figure R5. **Comparison between the pulse-induced metastable states in bulk crystals and thin flakes.** **a,** Normalized resistance as a function of time measured *in-situ* for both cooling (blue) and heating (red) branches for $EuTe_4$ bulk crystal. **b,** Similar results from exfoliated $EuTe_4$ thin flakes. Reproduced from Ref. 30 in the original manuscript.

Changes made:

We have added a paragraph to highlight the differences and novelty of our work before the last paragraph.

2) It is not experimentally supported that the resistance changes are due to only the reduction of CDW strength. In the manuscript, the author claimed that "electrical excitation induces disorder and potentially multiple domains, contributing to the observed sharp resistance drop." Yet in Fig.3c and d, from time stamp 0 to 1, the XRD measurements show essentially no reduction in either CDW intensity or out-of-plane correlation length, even though the resistance has already

dropped significantly. The current manuscript does not provide an explanation for this discrepancy, which weakens the argument that CDW weakening directly drives the resistance change. Alternative mechanism or additional evidence should therefore be considered to substantiate this interpretation.

We thank the reviewer for pointing out this critical issue. As we also discussed in our response to Reviewer #1 above, the CDW amplitude do not have a one-to-one correspondence to the resistivity. Upon electrical excitation, it possible that networks of metallic domain walls form in the material and provide conductive channels that effectively reduces resistivity. In this case, the CDW amplitude observed in ARPES and XRD, due to the different physical quantity and material depth probed, do not necessary scale together with the decrease in resistivity. However, when a significant amount of domain walls form the in the system, the measured CDW amplitude that is averaged over the sample start to show significant suppression [7,8].

Changes made:

We have added a discussion on other possible explanations for the driving mechanism of the metastable CDW phases. We also extended the discussion on the connection between ARPES, XRD and resistance measurements.

3) ARPES measurements at intermediate stages are needed. In Fig. 2, the authors present ARPES measurements only before and after the full sequence of electrical pulses. The rigid shift of the EDC before and after applying the electrical pulses was used as evidence for the suppression of the CDW gap in the metastable phase. However, ARPES measurement is missing at intermediate stages, unlike the XRD measurement in Fig. 3. If the resistance has already dropped significantly at a stage similar to time stamp 1, and if this drop indeed reflects partial suppression of the CDW order, one would expect a corresponding intermediate shift in the ARPES EDC. The absence of such measurements leaves a critical knowledge gap, and additional ARPES data would be necessary to clarify whether the electronic structure evolves stepwise in concert with the transport response. In addition, the two CDW q-vectors should be labeled in the constant-energy contours in Fig. 2. It is not clear how to compare the ARPES results in the current study to a previous study by the same authors (Fig. 3 of Ref. 27).

We appreciate this important point raised by Reviewer #2. Following the reviewer's suggestion, we performed additional *in situ* ARPES and transport measurements at intermediate stages of the voltage-pulse sequence. The main results are summarized in Fig. R3. As correctly noted by the reviewer, a gradual upward shift of the EDC leading edge is observed, which correlates well with the concurrent change in resistance. This consistency provides further evidence for a progressive suppression of the CDW gap in the metastable phase.

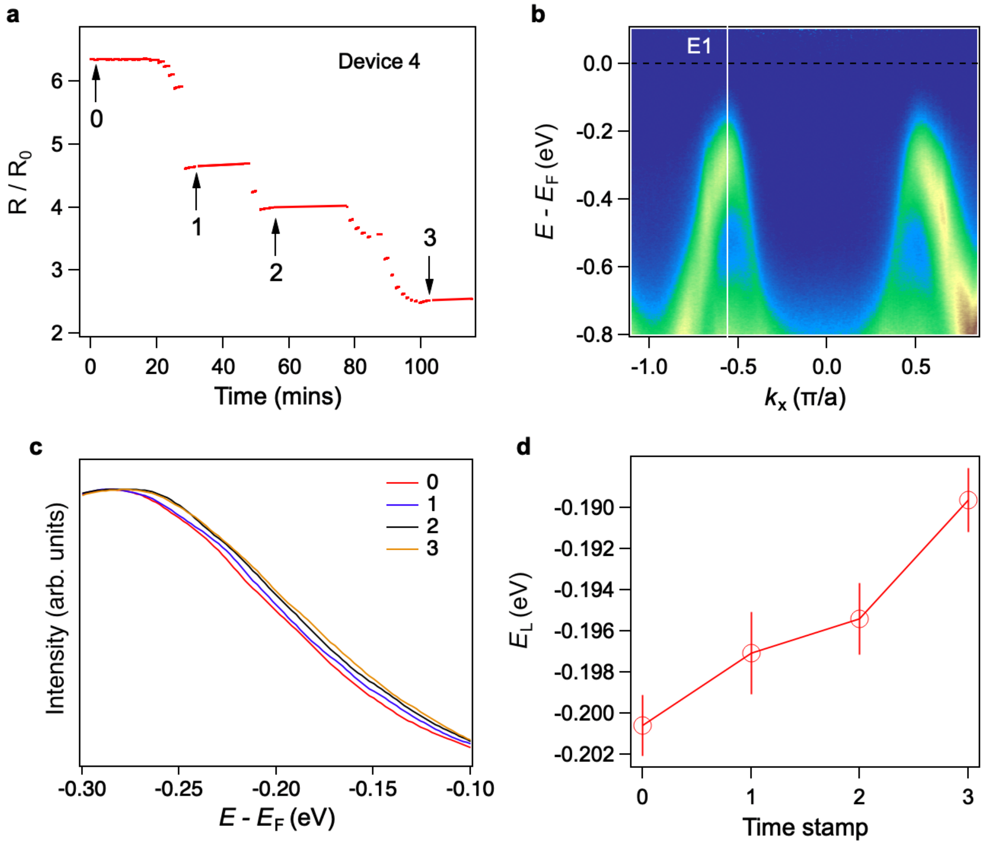

Figure R3. **Reproduced CDW gap suppression upon current pulse excitation in ARPES measurement on Device 4. a**. Concurrent resistance measurement upon pulse current excitation, showing decreasing resistance consistent to data presented in Fig. 2a. **b**. Energy-momentum cut of spectral function measured with ARPES on device 4, consistent with the spectrum in Fig. 2c. **c-d**. Energy distribution curves (**c**) and extracted leading-edge position (**c**) corresponding to the time stamps 0,1,2, and 3 in **a**, indicating a gradual upward shift of the band leading edge and thus a suppression of CDW amplitude.

Changes made:

We added a new section in the supplementary information for additional transport-ARPES measurement results. We also labeled the constant-energy contour in Fig. 2 with a CDW nesting vector. However, please note that it is impossible to differentiate the monolayer and bilayer CDW vectors in real ARPES measurements due to finite momentum resolution. We thus only labeled a q_{CDW} vector, consistent with our previous trARPES work [6].

4) The switching does seem to depend on the pulse duration, contrary to what the authors claimed. In Fig. 1c, the authors showed a voltage-dependent transport measurement, indicating a positive correlation between the applied voltage and the amount of resistance drop. However, in Fig. 4b,

the magnitude of the resistance change also appears to grow systematically with increasing pulse duration, suggesting that both parameters, field strength and pulse duration, affect the transition. What the authors should do is two systematic studies: first, study the resistance after switching as a function of the applied voltage, fixing the pulse duration; second, study the resistance after switching as a function of the pulse duration, fixing the applied voltage. Without such systematic studies, no clear conclusion can be made.

We sincerely apologize for the misleading wording in the original manuscript. We fully agree with the reviewer that the observed metastable states exhibit a non-negligible dependence on pulse duration (see Fig. 1c), especially for longer pulses (see Fig. 4b). This observation suggests that, in addition to electric-field effects, thermal contributions associated with Joule heating cannot be ruled out in the formation of these metastable states. We have corrected the relevant statements accordingly in the revised manuscript.

With respect to the transport measurements, we agree that an ideal approach would be to perform control experiments that independently and systematically vary the electric field and the pulse duration. While experimental constraints prevent a complete separation of these two parameters in all regimes, our measurements were designed to partially disentangle their respective roles. Specifically, in the first regime of Fig. 1c (yellow region), we track the evolution of the resistance as a function of applied voltage while keeping the pulse duration fixed at 10 μs.

Starting from pulse #3, the measurements shown in Fig. 2 are carried out by increasing the pulse duration while keeping the voltage approximately constant. We note that, to minimize the risk of sample damage or circuit failure, two preparatory pulses are applied to gradually increase the voltage to its maximum value. In addition, due to limitations of the maximum output current of the pulse generator, the achievable voltage decreases as the sample resistance is reduced. As a result, the voltage cannot be held strictly constant throughout the pulse sequence in Fig. 2. These experimental constraints are now clarified in the revised manuscript.

Changes made:

We extended the discussion on experimental constraints in the revised manuscript. We have also provided a more careful discussion on the pulse duration dependence and the conclusion of electric-field-driven CDW metastable states.

5) In absence of the analysis separating the effects due to applied voltage and due to pulse duration, thermal contributions cannot be ruled out, especially given the well-documented large thermal hysteresis in $EuTe_4$. Both higher voltage amplitudes and longer pulses can transiently heat up the sample, which could independently lead to a decrease in resistance. One question is whether the authors tried a “heating” pulse. For example, one can apply such a heating pulse by applying a

high repetition rate or cw laser. I note that an electrical pulse duration of micro-seconds is much beyond the typical time scale for lattice to be heated up.

Following the reviewer's suggestion, we provide a quantitative estimate of the possible Joule-heating effects associated with voltage pulses of different durations.

For simplicity, assuming that all electrical energy delivered by the voltage pulse is converted into heat within the sample before dissipating to the environment (neglecting the enthalpy associated with phase transitions and any possible energy emission at early times after current injection) and the entire sample is heated uniformly, the resulting temperature increase ΔT of the sample can be estimated as

$$\Delta T = \frac{E \times M}{C_p \times \rho \times V}$$

where E is the electrical energy of the voltage pulse, M = 662.4 g/mol is the molar mass, C_p=125 J/(mol·K) is the molar heat capacity, ρ = 6.768 g/cm^3 is the mass density, and V is the volume of the $EuTe_4$ bulk crystal between the electrodes (assuming the entire sample is homogenously heat up).

Unlike Refs. 29 and 30, which focus on thin flakes, our measurements are performed on bulk single crystals. A typical sample volume is approximately 2×0.5×0.03 mm^3, corresponding to $V \approx 3\times10^{-5}$ cm^3. Using these parameters, the effective heat capacity of the probed crystal volume is estimated to be on the order of 3.83×10^{-5} J/K.

As a representative example, we consider Device 2 in the heating branch at 300 K, with resistance $R \approx 500\ \Omega$, applied voltage U = 12 V, and pulse duration τ. The total electrical energy delivered by a pulse is estimated as

$$E = U^2\tau/R$$

For a pulse duration of 10 μs, this yields $E \approx 2.88\times10^{-6}$ J, corresponding to a temperature increase of $\Delta T \approx 0.075$ K. Even for a much longer pulse duration of 1 ms, the estimated temperature increase is only ~7.5 K, which corresponds to a resistance change of ~8% (see Fig. 1b). This is below the observed drop of more than 50% (see Fig. 2a).

From the above analysis, we conclude that Joule heating is unlikely to be the dominant factor for short voltage pulses, particularly in the microsecond regime. However, it is also important to clarify that we cannot completely rule out thermal contributions, especially for longer pulses. Consistent with this consideration, the sensitivity of the metastable states to pulse duration may indicate that both electronic and thermal effects contribute to the observed metastability. Accordingly, we do not explicitly exclude thermal effects in the main text.

Changes made:

We included a new section in the supplementary materials to include the above discussion on Joule heating.

6) The authors suggest that $EuTe_4$ could potentially be used as non-volatile memory devices. However, based on the proposed switching mechanism of gradual evolution of domain sizes, one would expect intrinsic stochasticity in the domain-evolution process. If structural domain growth governs the resistance changes, then after thermal erasure, repeated application of identical voltage pulses should not necessarily lead to identical resistance states. The authors should provide repeatability tests demonstrating that the resistance switching is reproducible over multiple cycles under identical electrical conditions. Without such evidence, the suitability of $EuTe_4$ as a reliable memory medium remains unproven.

We thank the reviewer for the critical question. However, we believe the variation of specific CDW configuration does not affect the reproducibility of the resistivity change upon excitation.

We agree with the reviewer that the specific CDW configuration within the domain as well as the specific distribution of the domains upon electrical excitation are probabilistic. However, the resistivity of the device is eventually governed by the charge density wave amplitude averaged over the sample and the domain wall formation across the sample. Thus, even though the specific CDW configuration can vary, the resistivity change remains reproducible upon excitation as the average CDW amplitude and correlation length predominantly depend on the pulse energy of the excitation pulse. This is similar to the case of 1*T*-TaS_2, a CDW system that is widely recognized as memory device candidate, given the first-order nature of CDW phase transitions and pulse-induced domain dynamics [9].

Changes made:

We made a note on the reproducibility at the end of the third to last paragraph.

7) Fig. 4d lacks appropriate labeling and explanation. What parameter space are these cubes plotted in? xyz space? kx-ky-kz space? It is not clear at all.

We thank the reviewer for pointing out the ambiguity of the schematics. The picture is meant to present the domain dynamics in real space. As shown in Fig. R4, the blue color represents the domains with ground state CDWs, while other colors represent metastable domains, with each distinct color representing a distinct CDW stacking configuration.

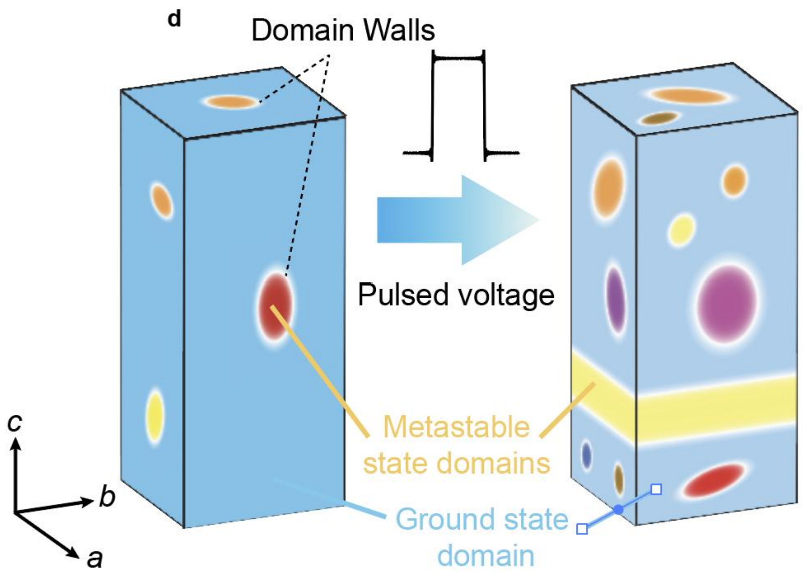

Figure R4. ***Revised Fig. 4d in the main text.*** **Schematics of voltage-induced metastable CDW states.** The cuboids indicate the domain dynamics in real space, labeled with crystalline axes. The area with cyan color represents the ground state, and areas with other colors represent metastable CDW domains, where different colors indicate different metastable phases with distinct relative phases between the Te layers, as color-coded in the table in **c**. The thin white boundaries indicate domain walls between distinct CDW domains. Upon pulsed electrical excitation, the ground state CDW amplitude is suppressed, and metastable CDW

Changes made:

We have labelled spatial coordinates with crystalline axes. We have also added more detailed explanation in the caption as shown above in Fig. R4.

8) The paper only shows data in the heating cycle. What happens to the resistance switching in the cooling branch of the hysteresis loop (see Ref. 30)?

The data in cooling branch was presented in the supplementary materials.

9) Resistances in the metastable states are still changing over time, even though it is slow. Note that if this is for quasi-permanent information storage, this would still be a big problem. The authors showed that the resistance changes by 7% in a few hours. What happens if we let the materials idle for a few days or a few weeks? Will the resistance completely recover to the value before switching?

We thank the reviewer for pointing out the critical question that affects the potential memory device applications. To answer reviewer's question, we performed additional resistance measurement over a timespan of over three days. As shown in Fig. R6, after applying current

pulses that reduce the resistance to around 70% of the original value, we can clearly observe an extremely slow logarithmic recovery. Extrapolating from the logarithmic fitting, the time for 60% resistance recovery (to 90% of the original value) is on the order of magnitude of 10^{11} hours (millions of years). We thus believe the switching can be considered as quasi-permanent.

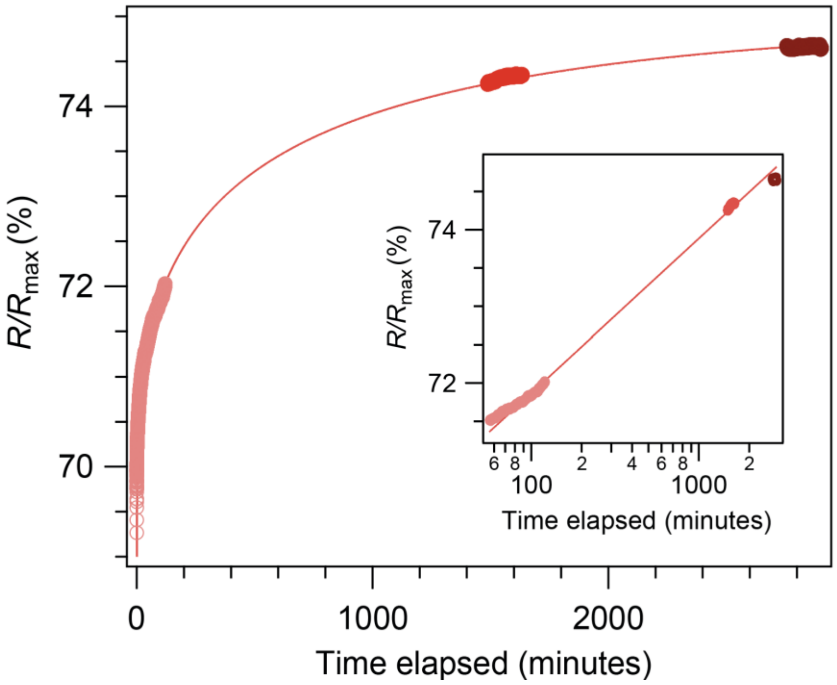

Figure R6. **Recovery dynamics of resistance after pulsed current excitation.** Time evolution of resistance over the three days after applying current pulses. Inset: Same data plotted in logarithmic scale. Inset: resistance recovery plotted in log scale, featuring logarithmic recovery dynamics.

Changes made:

We added a new section in the supplementary materials discussing the recovery time scale of resistance.

Reviewer #3 (Remarks to the Author):

The manuscript by Lv et al. reports the observation of electrically driven multistage metastable states in $EuTe_4$ at room temperature, characterized by discrete resistivity plateaus and high sensitivity to the applied electric field. By integrating photoemission spectroscopy, X-ray diffraction, and transport measurements, the authors attribute these states to electric-field-induced

switching between out-of-plane charge-density-wave (CDW) phases within the moiré superstructure.

The reported effect is interesting and may hold potential for memory device applications. The data analysis is technically sound in general.

We thank the reviewer for acknowledging the potential interest for the community and the scientific rigor of our work.

However, I have several major concerns regarding the novelty of the findings relative to very recent literature, as well as the interpretation of key experimental results, which must be addressed before the manuscript can be considered for publication.

1. Two preprints/papers (Nature Communications 15, 8937 (2024) and arXiv:2412.13094) also reported nonvolatile metastable states in $EuTe_4$, induced by light and electric field, respectively. The authors must explicitly clarify the principal advance of the present manuscript compared to these works. A more detailed and focused discussion highlighting the distinct mechanism (e.g., the specific role of out-of-plane CDW switching in a multi-stage manner), or new insights offered here, should be added to the introduction and/or discussion.

We thank Reviewer #3 for raising this important point.

As noted in our manuscript, recent transport and optical studies have demonstrated optical and electrical switching of hidden, nonvolatile CDW states in $EuTe_4$ flakes**,** opening the door to understanding metastable behaviors in this room-temperature moiré superstructure (Refs. 29, 30).

The present work, however, is fundamentally distinct from these studies in platform, methodology, focus, and observed phenomena.

First, platform. Refs. 29 and 30 focus on exfoliated $EuTe_4$ flakes with lateral dimensions of tens of micrometers and thicknesses of several tens of nanometers. In contrast, our study investigates bulk single crystals with typical dimensions of ~$2\times0.5\times0.05$ mm^3. This difference in dimensionality is crucial and leads to qualitatively different metastable behaviors.

Second, methodology and focus. Ref. 29 focuses on light-induced metastable states, while Ref. 30 relies exclusively on transport measurements to reveal electrically switched states. In contrast, as illustrated in Fig. 2, our work uniquely combines *in situ* transport with ARPES and XRD to investigate current-induced metastable states. This integrated approach enables direct probing of CDW order parameters under voltage perturbation, and naturally shifts the focus of the present work towards revealing the nature and microscopic origin of the metastable states. We emphasize that performing in situ transport together with ARPES and XRD is technically nontrivial, requiring careful control of temperature, vacuum conditions, grounding, and data acquisition. To the best of our knowledge, this is the first work to integrate *in situ* transport, ARPES, and XRD within a single experimental framework.

Third, and most importantly, the observed phenomena are qualitatively different.

As correctly noted by the reviewer, metastable behaviors in bulk crystals and flakes differ markedly (Fig.R5):

3. **Cooling branch:** For bulk crystals, the resistivity shows little change under voltage driving. In contrast, for flakes, a single voltage pulse of 2 V or an ultrafast optical pulse with fluence exceeding F~6.8 mJ/cm^2 can drive the system into hidden high-resistance metastable states.
4. **Heating branch:** As correctly noted, metastable states in both bulk crystals and exfoliated flakes can be electrically tuned, but their responses to voltage pulses are distinct. In bulk crystals. In bulk crystals, a sequence of voltage pulses with increasing amplitude (up to 12 V) drives the system into multiple metastable states characterized by substantially reduced resistivity (exceeding a 50% drop). In contrast, for flakes, voltage pulses below 2 V induce metastable states with lower resistivity, whereas for voltages exceeding 2 V the system transitions into hidden high-resistance states.

These striking differences directly reflect the novelty and significance of the present work. As discussed in the main text, due to the quasi-two-dimensional nature of the Te layers, the in-plane couplings are much stronger than the out-of-plane couplings. Consequently, the application of an electric field predominantly modifies the out-of-plane periodicity of the monolayer and bilayer CDWs.

For simplicity, as illustrated in Fig. 4c, we assume that the intra-bilayer phases are locked ($\theta_3 = \pi$) and that the in-plane coherence length is effectively infinite. Under these assumptions, considering a 2c (~3.1 nm) unit yields four classes of out-of-plane CDW configurations: (0,0), (0,π) , (π,0), and (π,π).

For thin flakes with typical thicknesses of several tens of nanometers, there are on the order of ten such units along the c-axis. A voltage pulse can therefore drive the entire stack into one of the metastable configurations, naturally explaining the emergence of hidden high-resistance states. In contrast, for bulk crystals with thicknesses of tens of micrometers, there are on the order of 10^4 units along the c direction. It is therefore far more difficult to coherently align all units into a single metastable configuration. This naturally explains the observed distinct metastable behaviors of bulk crystals, especially the emergence of multiple metastable states in the heating branch and the absence of hidden high-resistance states.

To summarize, our work is neither in contradiction with nor a reproduction of the findings in Refs. 29 and 30. Rather, it provides a complementary perspective. Specifically, the current work represents the first systematic study of current-induced metastable CDW phenomena in bulk EuTe4 and the first to directly track voltage-driven evolution of CDW order parameters using a combined transport–ARPES–XRD approach. We therefore strongly believe that this study meets the scope and significance criteria of *Nature Communications*.

Changes made:
We have added one paragraph to highlight the differences and novelty of our work.

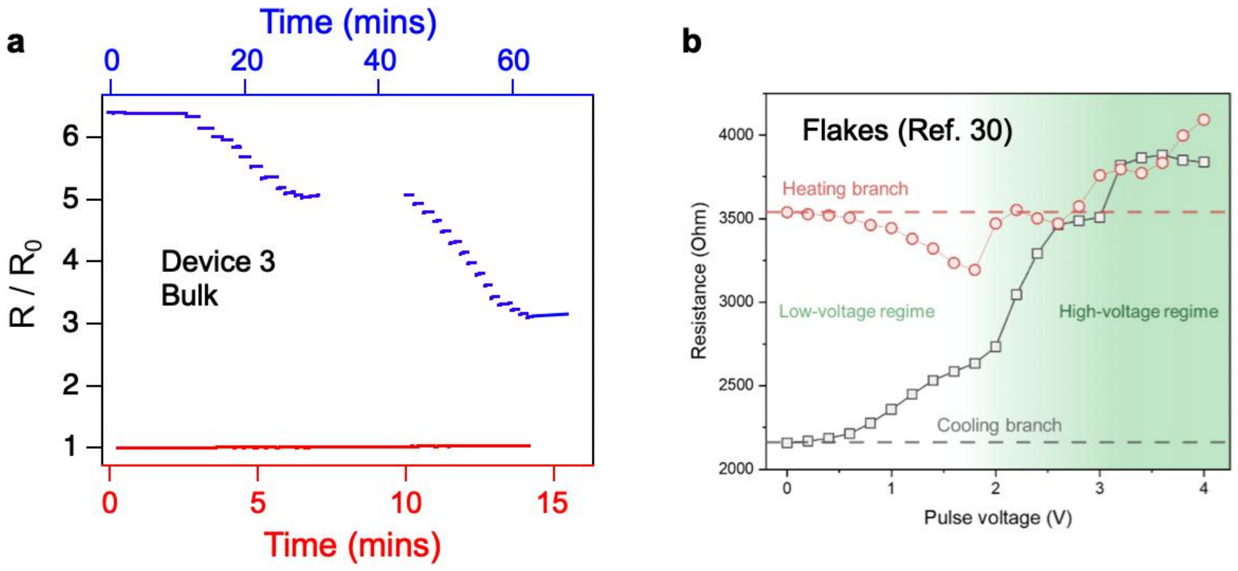

Figure R5. **Comparison between the pulse-induced metastable states in bulk crystals and thin flakes.** **a,** Normalized resistance as a function of time measured in-situ for both cooling (blue) and heating (red) branches for $EuTe_4$ bulk crystal. **b,** Similar results from exfoliated $EuTe_4$ thin flakes. Reproduced from Ref. 30 in the original manuscript.

2. Is the electric-field-induced resistance switching observable in the normal phase of $EuTe_4$ (i.e., above the CDW transition temperature), or is it exclusively enabled by the presence of the CDW order? A control experiment above the transition temperature would be crucial to establish the essential role of the CDW state and strengthen the proposed mechanism.

Unfortunately, the CDW transition temperature of $EuTe_4$ is way above the highest temperature available for most, if not all, existing ARPES and XRD setups. There was one transport study that reported indirect evidence of a transition temperature ~ 652 K and suggested a metallic state above that temperature. In the case of metallic system, with or without CDWs, it would be nearly impossible to differentiate pulse-induced metastable states from resistivity, as previously exemplified in the study of 1*T*-TaS_2 [9] The discussion of potential resistive memory device is not well defined in that case and thus is beyond the scope of the present work. On the other hand, we would like to note that the large number of nearly degenerate CDW states in $EuTe_4$ is a crucial factor for programable metastable states and thus for resistive memory device.

3. The in-situ ARPES data in Fig. 2d, intended to demonstrate CDW melting via a band shift after voltage pulses, does not show a visually clear energy shift. Furthermore, only one energy distribution curve (EDC) is presented in Fig. 2e. Could the authors provide a more quantitative

analysis (e.g., momentum-integrated EDCs) to unequivocally demonstrate a rigid shift or CDW gap melting?

Following the reviewer's suggestion, we integrated 50 EDCs in the vicinity of the valence-band maximum and extracted the leading-edge position of the integrated EDC. As shown in Fig. R7, the integrated EDC exhibits a clear upward shift of 9 ± 2 meV, in quantitative agreement with the result shown in Fig. 2e (Fig. Rb). This independent analysis robustly supports the partial melting of the CDW induced by current injection, rather than a fitting- or thermal noise-related artifact.

Importantly, the upward shift of the EDCs is consistently reproduced in Device 4. The reproducibility across different devices rules out sample-specific effects and further confirms that the observed energy shift originates from a current-induced weakening of the CDW amplitude.

Changes made:

We have updated Paragraph discussing ARPES results and added Section V on additional ARPES data and analysis to the Supplementary Materials.

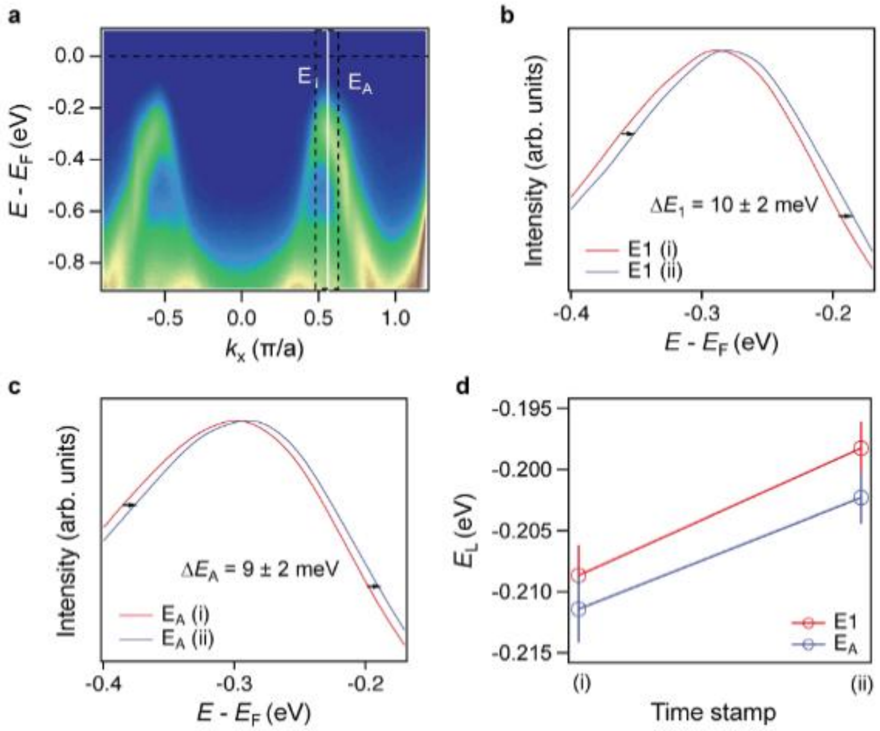

Figure R7. **Robustness against the choice of momentum window for energy distribution curves study.** **a,** Energy-momentum dispersion cut of CDW-gapped band, same as Fig 2b in the main text. **b**, Energy distribution curves (EDCs) cut along the white solid line (E_1) in a, same as Fig. 2e. **c**, EDCs before and after

pulse excitation acquired by integrating over the dashed momentum window in **a**. **d**, The leading edge positions before and after pulse excitation extracted from the EDCs with (E_A) and without (E_1) integrating over the momentum window. Showing consistent energy shifts, up to the systematic uncertainty of the measurement, in the band's leading edge.

4. Please state the energy resolution and measurement temperature of the ARPES experiment, as these parameters are critical for interpreting subtle spectral changes.

We appreciate this close inspection. The APRES measurements are done at 300K and the combined the combined (beamline and analyzer) experimental energy resolution was 6 meV and 16 meV for energy-momentum cuts and Fermi surface maps, respectively (See Methods Section). According to the supplemental material of the following ARPES paper on cuprates (*Nature* **450**, 81 (2007), https://doi.org/10.1038/nature06219), a 2 meV energy gap can be resolved with a 5 meV energy resolution. Therefore, the 6 meV energy resolution in our measurements is sufficient to resolve the 10 meV shift in $EuTe_4$.

Change made:
Following the reviewer's suggestion, we have added the relevant measurement temperature to the caption of Fig. 2.

5. The X-ray diffraction data in Fig. 3 shows a significant change in resistivity after the first voltage pulse, yet the corresponding diffraction peak intensity and full width at half maximum (FWHM) appear nearly unchanged. Are there additional quantitative data (e.g., integrated intensity and FWHM fitting results) to substantiate the claimed evolution?

As we also discussed in our response to reviewers above, the CDW amplitude and correlation length do not have a one-to-one correspondence to the resistivity. Upon electrical excitation, it possible that a small number of metallic domain walls, where CDW amplitude is locally suppressed, form in the material and provide conductive channels that effectively reduces resistivity. In this case, the CDW amplitude observed in ARPES or XRD does not necessary scale with the decrease in resistivity. The change in correlation length may also be within the resolution of X-ray diffraction. However, when a significant amount of domain walls form the in the system, the measured CDW amplitude that is averaged over the sample start to show significant suppression [7,8].

Change made:
Following the reviewer's suggestion, we have added the relevant measurement temperature to the caption of Fig. 2

6. The authors conclude in page 8 that "the application of electric fields predominantly modifies the out-of-plane periodicity... while the in-plane periodicity remains preserved." What would be

the effect if the electric field were applied along the out-of-plane direction (c-axis)? A discussion on the expected anisotropy based on the proposed mechanism is necessary to complete the physical picture.

We appreciate the interesting question. We would like to first stress that the robustness of in-plane periodicity is locked due to the unique joint lock-in phenomenon in $EuTe_4$. As we discovered in our previous study, the two CDWs in monolayer and bilayer Te sheets forms a pair of jointly commensurate CDW. This joint lock-in makes the *q*-vector extremely robust, not only against temperature, but also against optical excitation, as we revealed in a more recent ultrafast pump-probe study, as well as electrical excitation as we discuss in the present work. As a result of the joint lock-in, the energy barrier of changing *q* vectors (> 1eV) is orders of magnitude higher than that of shifting the relative interlayer phases (~10 meV).

Given all the external excitations exhibited in $EuTe_4$, both optical and electrical, being predominantly incoherent, we do not expect an out-of-plane electric field excitation have a qualitatively different effect unless a high enough field that goes beyond the energy scale of the present study is applied. We would also like to note that experimentally it would be nearly impossible to perform ARPES or XRD measurements with electrodes for applying out-of-plane electric field.

Change made:
We highlighted the robustness of in-plane *q*-vectors against thermal and optical excitations due to joint lock-in in the introduction paragraphs.

Reviewer #4 (Remarks to the Author):

I co-reviewed this manuscript with one of the reviewers who provided the listed reports. This is part of the Nature Communications initiative to facilitate training in peer review and to provide appropriate recognition for Early Career Researchers who co-review manuscripts.

References

[1] Lv, B.Q., Su, Y., Zong, A. *et al.* Large moiré superstructure of stacked incommensurate charge density waves. *Nat. Mater.* (2025)
[2] Oh, K.H., Su, Y,. Ning, H. et al. Joint commensuration in moiré charge-order superlattices drives shear topological defects. *arXiv:2509.16493*
[3] Dong, W.-H., Duan, W., Xu, Y., Tang, P. Multiple Layer-Selective Polar Charge Density Waves in $EuTe_4$. *arXiv:2602.09855*

[4] Jiang, Y., Chen, Z., Han, Y. et al. Electron ptychography of 2D materials to deep sub-ångström resolution. *Nature* **559**, 343–349 (2018)
[5] Ai, F., Shpyrko, O., Lomakin, V. Real-Time 3D Coherent X-Ray Diffraction Imaging *Phys. Rev. Lett.* **134**, 236202 (2025)
[6] Lv, B.Q., Zong, A. *et al.* Coexistence of Interacting Charge Density Waves in a Layered Semiconductor *Phys. Rev. Lett.* **132**, 206401 (2024)
[7] Orenstein, G., Duncan R.A. *et al.* Dynamical Scaling Reveals Topological Defects and Anomalous Evolution of a Photoinduced Phase Transition *Phys. Rev. X.* **15**, 031058 (2025)
[8] Zong, A., Kogar, A., Bie, YQ. *et al.* Evidence for topological defects in a photoinduced phase transition. *Nature Phys* **15**, 27–31 (2019)
[9] Vaskivskyi, I. et al., Controlling the metal-to-insulator relaxation of the metastable hidden quantum state in 1*T*-TaS_2. *Sci. Adv.* **1**, e1500168(2015)

Reviewer #1 (Remarks to the Author):

The authors have addressed all my concerns. I recommend the manuscript for publication.

We thank the reviewer again for reviewing this work.

Reviewer #2 (Remarks to the Author):

The authors have done a commendable job in responding to questions from me and other reviewers. The insight about how the switching dynamics is different for bulk samples and for thin flakes with 10s of nm thicknesses is very important. I recommend publication of this work.

We thank the reviewer for acknowledging the improvement.

Reviewer #3 (Remarks to the Author):

The authors have performed additional data analysis, and tried as much as possible to address the question regarding the nolvety of their work compared to two recent works. Although the novelty is not very obvious, this work has included more detailed measurements, providing useful information for understanding the driven metastable phase in EuTe4.

We thank the reviewer for acknowledging the novelty of the work.

Reviewer #4 (Remarks to the Author):

I co-reviewed this manuscript with one of the reviewers who provided the listed reports. This is part of the Nature Communications initiative to facilitate training in peer review and to provide appropriate recognition for Early Career Researchers who co-review manuscripts.

We thank the reviewer again for co-reviewing this work.
